# Supplementary material for: Barley Yellow Dwarf Virus Influences Its Vector’s Endosymbionts but Not Its Thermotolerance
Source: Microorganisms. 2023 Dec 19;12(1):10. doi: 10.3390/microorganisms12010010 (PMC10819152; doi:10.3390/microorganisms12010010)
Supplement: Supplementary file 1 [file microorganisms-12-00010-s001.zip › microorganisms-2743060-supplementary.pdf]

Supplementary material

Methods

Comparison of genomic backgrounds

We compared the genomic background of our focal *R. padi* strain (OAT_02) against strains sampled in the Australian states of Victoria (*n* = 7), New South Wales (*n* = 2), and South Australia (*n* = 2), as well as the North American strain from Porras, Navas [1]. For all Australian *R. padi* strain, we extracted genomic DNA from pools of 30 individuals using Qiagen's DNeasy® kit. Whole-genome library construction was performed by Novogene (Novogene, Hong Kong, China) with sequencing performed on an Illumina HiSeq paired end 150 bp sequencing. For the North American strain, we downloaded single end transcriptome reads from NCBI (SRA Accession, SRR3203855). Reads were trimmed using *fastp* to trim for quality and residual adapter sequences to retain a mean quality of 20 and a minimum length of 90 bp [2].

Trimmed reads were mapped to the *R. padi* reference genome archived on GenBank (Genome Accession GCA_020882245.1). Whole-genome reads for the Australian samples were mapped with *bowtie2* [3]. Because there are currently no gene annotations associated with this reference, we curated our own set of annotations to facilitate mapping of the North American strain transcriptome. Repetitive elements in the genome >1000 bp were identified against the Insecta repeats in the RepBase (edition 20181026) of *RepeatMasker*. The identification was performed using *RepeatMasker* v4.1.2-p1 [4] with the NCBI Blast search algorithm. *De novo* identification of transposable elements (TEs) was performed using RepeatModeler [5]. The repeat-masked genome assemblies were submitted to the online tool *Helixer* [6] for genome structure annotation under the default parameters. Functional annotation was performed by blasting the proteins against the *EggNOG* v5.0 [7] database using *eggNOG-Mapper* [8]. The geneset proteins were also assessed by *BUSCO* based on the insecta_odb10 database. The program *gffread* was used to manipulate annotation files

[9]. After obtaining annotations, the North American strain transcriptome was mapped using *hisat2*. We used *samtools* to filter for mapping quality of 30, to sort, and to deduplicate, mapped reads. Variants were called using *freebayes* with a minimum mapping quality of 20, a minimum alternate allele count of 2, and pooled continuous allele frequencies in parallel on separate chunks of the genome [10, 11]. *Bcftools* was used to merege results. *Vcftools* was used to filter for SNPs, first applying a minimum variant quality of 20, then for minimum depth of 20, no missing data, and a minor allele count of 2 [12, 13]. Finally, only SNP variants were retained for downstream analysis.

Comparison among clones were performed in R. SNPs were imported using the *genomalicious* package [14]. The R package *data.table* and functions from *tidyverse* were used to manipulate the SNP data table, and to further filter loci so they only occurred on the four assembled chromosomes (not unassigned contigs) and were spaced at least 1,000 bp apart [15]. We estimated pairwise differentiation across all sample pairs with the Δ_D statistics using the *IDIP* function from the *HierDpart* package, available through GitHub [16]. The Δ_D statistic is analogous to F_{ST}, in the that is provides an estimate of the proportional differentiation among samples, but it is not subject to the same biases in heterozygosity or to any demographic assumptions. Hence, the Δ_D statistic is a more appealing measure of genetic differentiation among asexual aphid strains. Visualisations were performed using *ggplot2* [17].

Results

Our linear mixed model suggested no significant covariance existed between the relative density of BYVD and *Rickettsiella* (Fig. S1: $\chi 2$ = 0.99, d.f.= 1, p=0.32) or *Buchnera* (Fig. S1: $\chi 2$ = 0.27, d.f.= 1, p=0.61) density. The marginal R^2 for the model was low (0.049),

suggesting that, on an individual aphid level, BYDV density was not strongly associated with either *Buchnera* or *Rickettsiella* density.

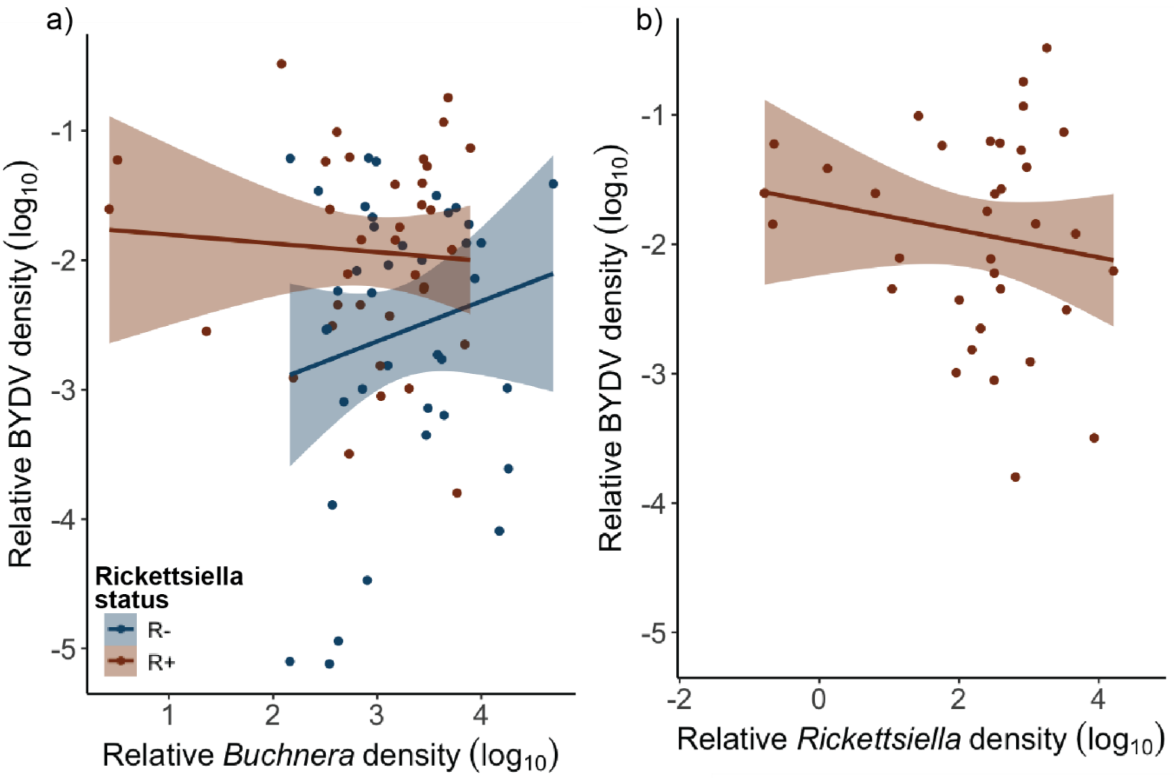

Figure S1**.** The association between the relative density BYVD density and *Buchnera (a) and Rickettsiella (b)* across individual *R. padi.* Lines show linear lines of best fit and shaded areas represent 95% Confidence Intervals. Note: the relative densities are plotted on a log scale.

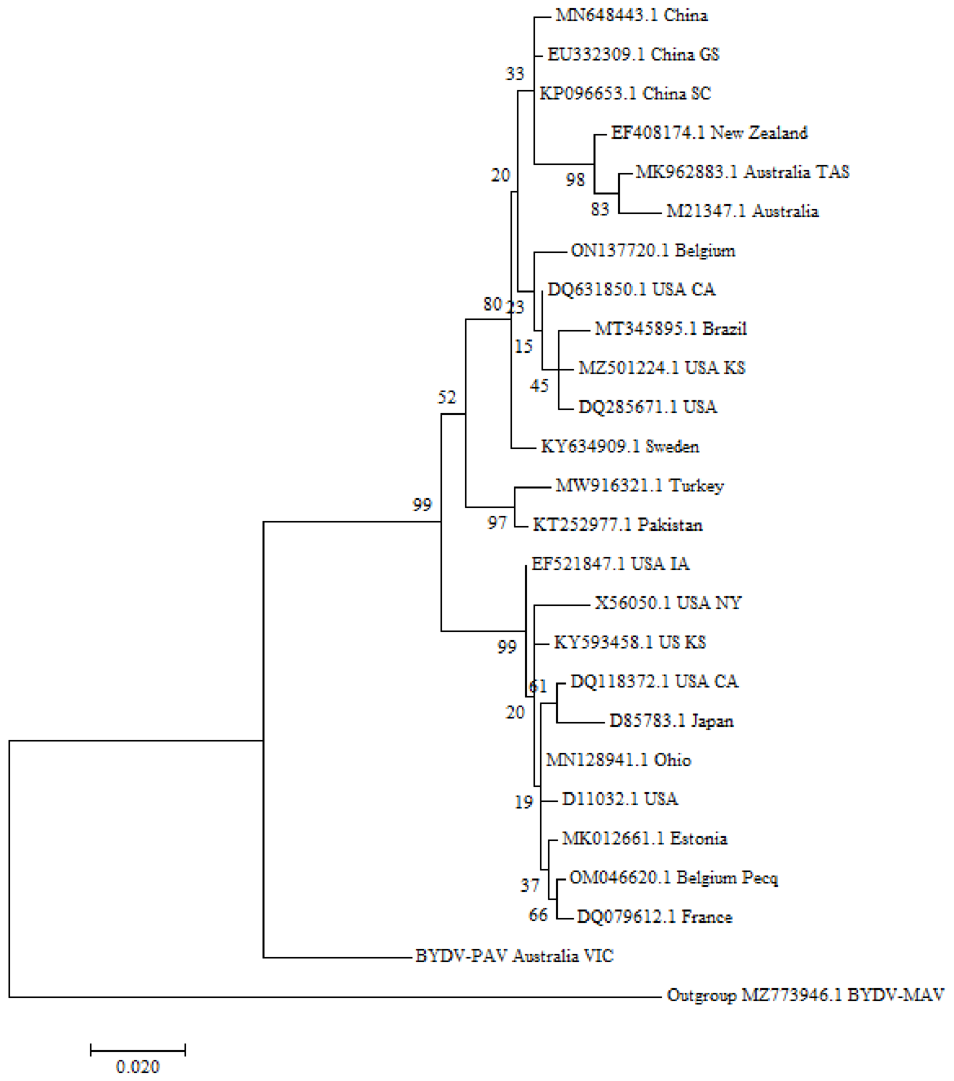

Figure S2. Phylogenetic analysis based on coat protein gene variation. Maximum-likelihood trees were constructed via Kimura-2 parameter model using MEGA 11. Numbers at branches represent bootstrap values of 1000 replicates. Sequences from different isolates of BYDV-PAV were selected from GenBank for comparison, with BYDV-MAV included as an outgroup.

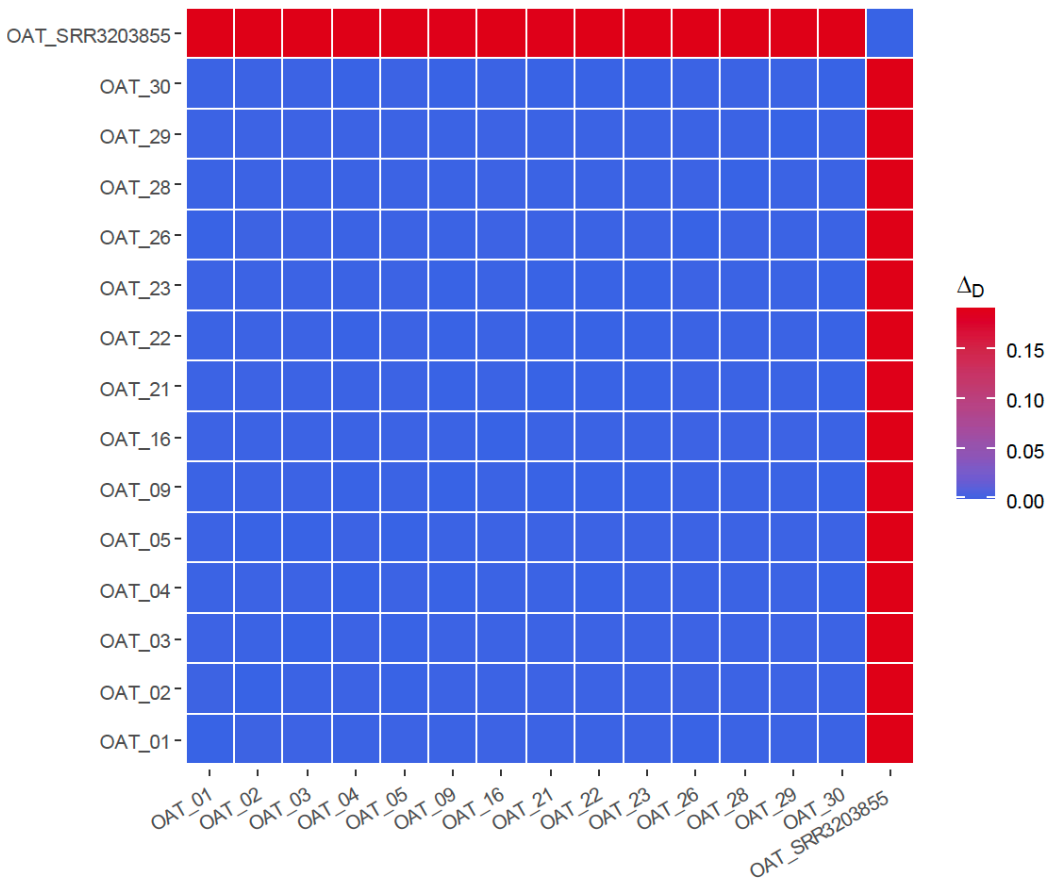

Figure S3. Heat map of pairwise genetic differentiation of the focal clone used in our study (OAT_02) among other Australian and American *R. padi* clones.

References

1. Porras, M.F., Navas C.A., Marden J.H., Mescher M.C., De Moraes C.M., Pincebourde S., et al. Enhanced heat tolerance of viral-infected aphids leads to niche expansion and reduced interspecific competition. Nature Communications. 2020;**11**,*1*,1184.

2. Chen, S., Zhou Y., Chen Y., Gu J. fastp: an ultra-fast all-in-one FASTQ preprocessor. Bioinformatics. 2018;**34**i884-i90.

3. Langmead, B., Salzberg S.L. Fast gapped-read alignment with Bowtie 2. Nat Methods. 2012;**9**,*4*,357-9.

4. Tarailo-Graovac, M., Chen N.S. Using RepeatMasker to identify repetitive elements in genomic sequences. Curr Protoc Bioinformatics. 2009;**Chapter 4**,*Unit 4.10*,1-14.

5. Flynn, J.M., Hubley R., Goubert C., Rosen J., Clark A.G., Feschotte C., et al. RepeatModeler2 for automated genomic discovery of transposable element families. Proc Natl Acad Sci U S A. 2020;**117**,*17*,9451–7.

6. Holst, F., Bolger A., Günther C., Maß J., Triesch S., Kindel F., et al. Helixer- de novo prediction of primary eukaryotic gene models combining deep learning and a hidden markov model. bioRxiv. 2023.

7. Huerta-Cepas, J., Szklarczyk D., Heller D., Hernandez-Plaza A., Forslund S.K., Cook H., et al. eggNOG 5.0: a hierarchical, functionally and phylogenetically annotated orthology resource based on 5090 organisms and 2502 viruses. Nucleic Acids Res. 2019;**47**,*D1*,D309-D14.

8. Huerta-Cepas, J., Forslund K., Coelho L.P., Szklarczyk D., Jensen L.J., von Mering C., et al. Fast genome-wide functional annotation through orthology assignment by eggNOG-Mapper. Mol Biol Evol. 2017;**34**,*8*,2115-22.

9. Pertea, G., Pertea M. GFF Utilities: GffRead and GffCompare. F1000Res. 2020;**9**.

10. Li, H., Handsaker B., Wysoker A., Fennell T., Ruan J., Homer N., et al. The Sequence Alignment/Map format and SAMtools. Bioinformatics. 2009;**25**,*16*,2078-9.

11. Li, H. A statistical framework for SNP calling, mutation discovery, association mapping and population genetical parameter estimation from sequencing data. Bioinformatics. 2011;**27**,*21*,2987-93.

12. Danecek, P., Bonfield J.K., Liddle J., Marshall J., Ohan V., Pollard M.O., et al. Twelve years of SAMtools and BCFtools. Gigascience. 2021;**10**,*2*.

13. Danecek, P., Auton A., Abecasis G., Albers C.A., Banks E., DePristo M.A., et al. The variant call format and VCFtools. Bioinformatics. 2011;**27**,*15*,2156-8.

14. Joshua, A.T., Cynthia R. genomalicious: serving up a smorgasbord of R functions for population genomic analyses. bioRxiv. 2019667337.

15. Wickham, H., Averick M., Bryan J., Chang W., McGowan L., François R., et al. Welcome to the Tidyverse. Journal of Open Source Software. 2019;**4**1686.

16. Gaggiotti, O.E., Chao A., Peres-Neto P., Chiu C.H., Edwards C., Fortin M.J., et al. Diversity from genes to ecosystems: A unifying framework to study variation across biological metrics and scales. Evol Appl. 2018;**11**,*7*,1176-93.

17. Wickham, H. ggplot2. WIREs Computational Statistics. 2011;**3**,*2*,180-5.
